# Is still effective massive allograft reconstruction in parosteal osteosarcoma of the distal femur? Review of the literature and advantages of newer technologies

**DOI:** 10.1186/s13018-024-04880-z

**Published:** 2024-07-08

**Authors:** Elisa Pala, Joele Canapeti, Giulia Trovarelli, Andrea Angelini, Pietro Ruggieri

**Affiliations:** Department of Orthopedics and Orthopedic Oncology, University of Padova, Via Giustiniani 3, 35128 Padua, Italy

## Abstract

**Purpose:**

Parosteal Osteosarcoma is a well-differentiated, low-grade bone sarcoma. It most commonly occurs in the third decade of life, usually in the distal femur. This study aims to perform a literature review about the types of reconstructions reported and to analyze the results of an updated technique of resection using custom-made 3D-printed cutting guides.

**Methods:**

We perform a systematic literature review about parosteal osteosarcoma, evaluating treatments, margins, local recurrence, complications, and functional results when available. We also report a case treated in our Center with a revisited technique introducing custom-made 3D-printed cutting guides.

**Results:**

We analyzed 12 studies with a total of 151 patients. The distal femur was the most frequently reported site (81.5%). After distal femur resection, reconstruction was performed with graft in most cases (48%), followed by prosthetic reconstruction (40%). Margins were wide in 85.5% of cases. The total incidence of local recurrence was 11%. Functional results were excellent in all cases, with a mean MSTS score of 86%. In our case, with the help of the jigs, the surgical technique was relatively easy, graft fusion excellent and fast, margins wide, and functional results excellent.

**Conclusions:**

In the literature, the most commonly used type of reconstruction after resection is biological with graft. Indeed, despite the increasing number of prosthetic reconstructions, the historical diaphysometaphyseal hemiresection and graft is still indicated in parosteal osteosarcoma of the distal femur. New technologies, such as the jigs we used, allow significant advantages during the procedure: reduce the resection and graft preparation time, allow a better match between components, and help to obtain safer margins, sparing as much bone as possible.

## Introduction

The first report on parosteal osteosarcoma was in 1951 by Geschicter and Copeland, who considered it a benign tumor of the surface called Parosteal Osteoma [1]. Thanks to numerous subsequent studies, it became clear that it was instead a low-grade malignant bone tumor and was officially defined as Parosteal Osteosarcoma (PO).

PO represents an uncommon malignant bone tumor, accounting for approximately 1% of primary bone tumors and 4% of all osteosarcomas [2]. It is a slow-growing malignant tumor with a female predominance (60%) and occurs in the third decade of life, thus in patients older than the median age of conventional osteosarcomas [3].

It usually arises from the bone cortical surface over the metaphyseal region. In 70% of cases, it is located at the posterior surface of the distal femur, followed by the proximal tibia and proximal humerus [4].

The clinical manifestation is represented by a painless mass lasting for an extended period, with a decreased range of movement of the adjacent joint. Dull pain and local tenderness are the second most common symptoms.

The tumor usually manifests on the X-rays as a lobulated mass protruding from the underlying cortex with a broad base attachment. It has an irregular pattern of mineralization, and its center is usually more radiodense than the periphery [4]. The extension into the medullary are better demonstrated by computed tomography and magnetic resonance imaging, which has been seen in 22% to 58% of patients. [6]

A characteristic cleavage between the tumor and the cortex can be seen in up to 65% of cases. The underlying bone cortex may be thickened or partially eroded, and the periosteal reaction is generally absent [6].

The treatment of PO is exclusively represented by surgery with wide-margin resection and reconstruction; chemotherapy and radiotherapy are not options to be considered.

Historically, the surgical approach was represented by the posterior diaphysometaphyseal hemiresection, according to Campanacci [7] but several types of reconstruction methods are still used in different countries: the most widely used is biological reconstruction with graft and screws; reconstruction with grafts and plates is also sometimes described; many cases of PO are also treated with segmental resection and reconstruction with a modular prosthesis [7–10].

This study aims to perform a literature review on the different surgical techniques for reconstructing the distal femur after resection of parosteal osteosarcoma to evaluate if the graft is still indicated and to describe how the newer technologies can improve the results.

## Material and methods

A systematic literature review was performed using PubMed and Google Scholar research libraries. The search terms used in combination were "parosteal osteosarcoma" and "distal femur." All manuscripts with full-text availability in English literature, published between 1990 and 2022, were analyzed. Exclusion criteria were: lack of complete information with only the abstract available, papers not reporting parosteal osteosarcoma, and papers reporting only parosteal osteosarcoma in sites different from the distal femur.

Two reviewers (JC, EP) independently double-screened all records to assess the manuscripts included in the study; a third reviewer (GT) checked all excluded records and resolved discrepancies.

The data extracted from the selected studies included treatments, margins, local recurrence, complications, and functional results when available.

We also reported a patient with distal femur PO treated in our Center by revisiting posterior diaphysometaphyseal hemiresection of the distal femur and reconstruction with a bone graft using custom-made 3D printed cutting guides.

The research has been performed under the Declaration of Helsinki. The patient gave written informed consent to be included in scientific studies upon admission to the hospital, and our Institutional Ethical Board Approval was obtained.

## Case report

**Fig. 1:**
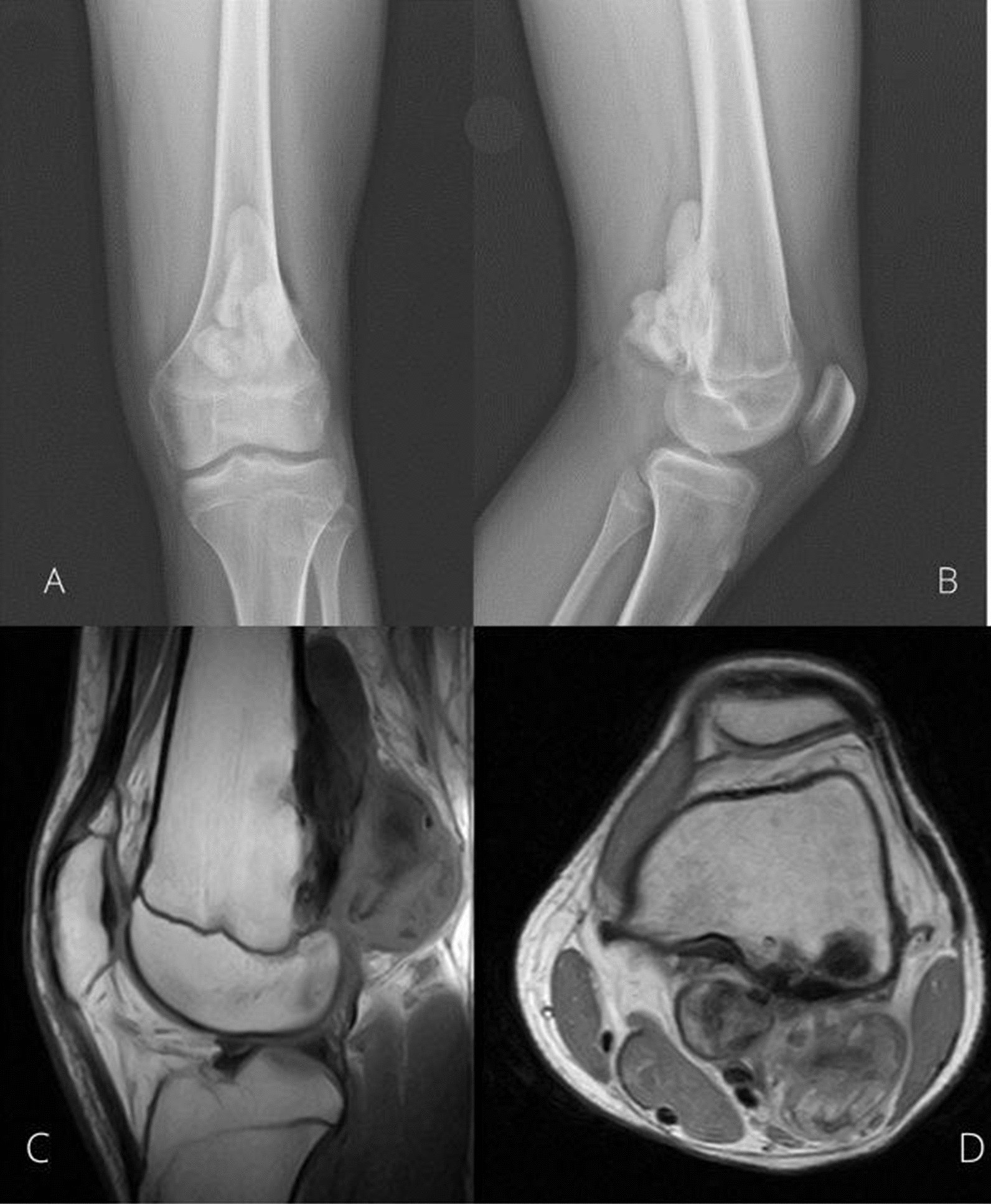
**A**, **B** Antero-Posterior and Lateral X-rays of the left knee show a lobulated, radiodense lesion protruding over the posterior cortex of the distal femur. It attaches to the underlying cortex with no invasion into the medullary bone. **C**, **D**: MRI: sagittal and axial scan of a T1-weighted image of the left distal femur of the patient. A protruding mass, with low signal intensity, arising from the posterior cortical of the femur can be seen. The medullary canal and the cruciate ligaments are not involved

We treated a 15-year-old boy with left distal femur PO. The radiological characteristics, the patient's symptoms, and the location of the lesion were pathognomonic for parosteal osteosarcoma, and there was no need for a biopsy to confirm the diagnosis (Fig. 1).

In the majority of radiologically typical case of evident low-grade lesions, biopsy can be omitted. Doubtful lesions should undergo biopsy to identify high-grade or dedifferentiated tumors [9, 20].

Historically, the surgical treatment for PO was the posterior diaphysometaphyseal hemiresection and reconstruction with bone graft, as reported by Campanacci et al. in 1984 [14].

This technique requires a double incision (one medial and one lateral) at the distal third of the thigh, prolonged beyond the joint spacing. The gastrocnemius and adductor major muscles are dissected 1–2 cm from their femoral insertion. The two surgical accesses can be joined to have an adequate view of the mass to be resected and a reasonable control of popliteal vessels and nerves [7].

We have reviewed the classic tumor resection and femoral reconstruction technique by introducing 3D-printed cutting guides for osteotomies.

3D printing is an additive manufacturing technique that allows to transform a digital model into a three-dimensional object. Three-dimensional models are obtained by processing digital radiological studies of patients, such as computed tomography (CT) scans, and when the virtual model has been obtained, it can be printed.

**Fig. 2:**
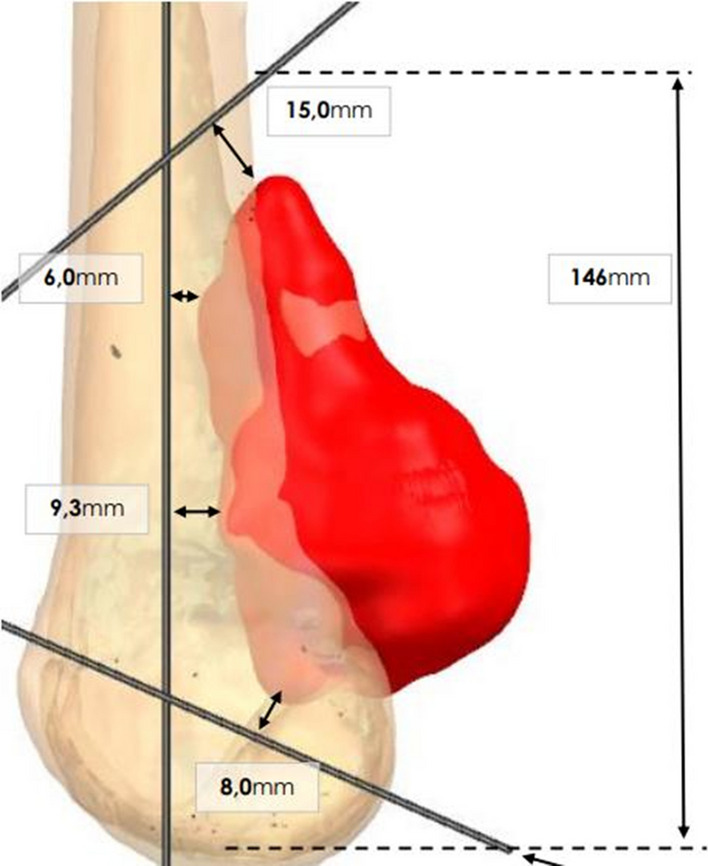
Resection planes based on CT scan. The coronal resection plane is posterior; the superior plane is tilted (40°), and the inferior plane is tilted (40°), aiming to preserve the posterior condyle

The planning was carried out with the collaboration of a team of engineers. First, a thin-layer (1 mm) CT scan of the host and graft distal femurs was performed (Fig. 2).

The images were then acquired in DICOM format (digital imaging and communications in medicine) and transferred to a specific reconstruction software (ProMade® platform, Lima Corporate, San Daniele del Friuli, Udine, Italy). The guides are in polyamide. These arrived non sterile and were stored at 0–50° in their protective closed packaging.

**Fig. 3:**
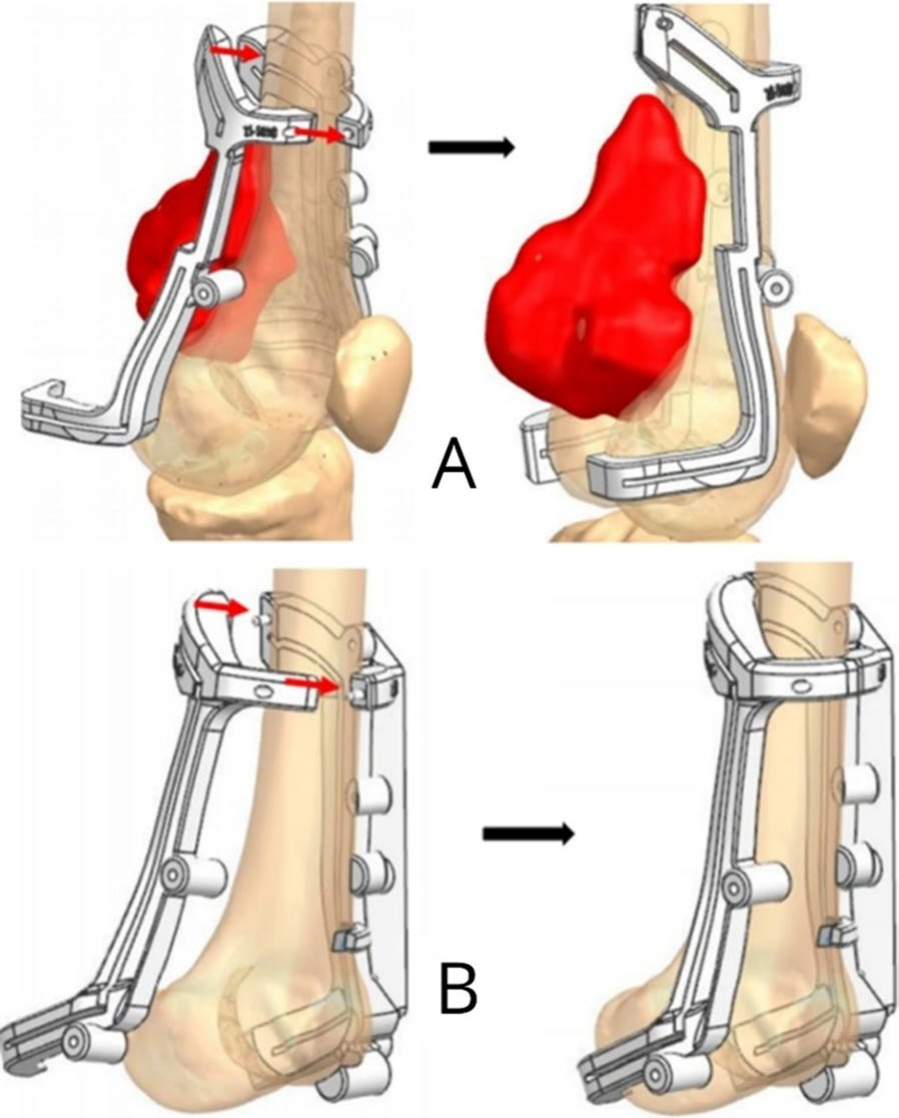
The resection jigs were one lateral and one medial, with the possibility of being connected and stabilized to the bone by pins. They allow following a precise and regular cut, previously planned on the CT scan. The jigs made for the native bone (**A**) and for the graft **B** are produced by the same system. This jigs for the graft perfectly fitted with the graft and have the same cutting line of the host bone

Based on these, the cutting planes for the patient and the graft are virtually established to obtain adequate wide margins (about 1 cm to each site except 0.8 cm in the distal part because we don’t have enough space), preserving the residual bone and trying to facilitate the reconstruction (Fig. 3).

Two weeks after the approval of the guide's design, these jigs and two phantom models of the femurs were sent to our Center.

According to the standard technique a double incision is required at the distal third of the thigh, prolonged beyond the joint spacing.

After isolating the muscles and the neuro-vascular bundle, the two lateral and medial surgical accesses can be joined, in order to have an adequate view of the mass to be resected and a good control on popliteal vessels and nerves. At this point we put the medial and lateral jigs on the femur and we cut the bone with a saw inserted in the hole of the jigs.

After the tumor resection we prepare the graft with the dedicated cutting guides.

**Fig. 4:**
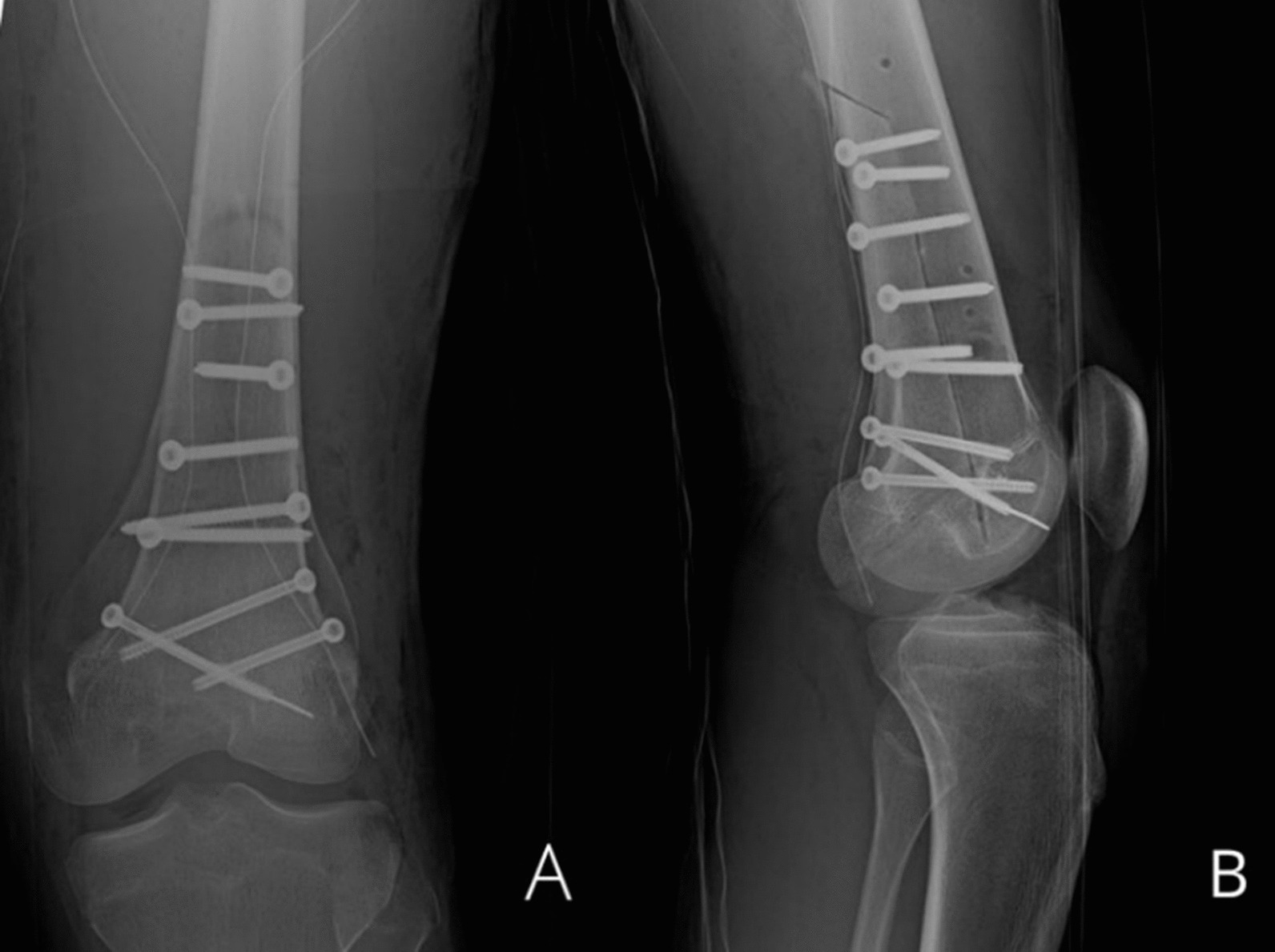
Antero-Posterior and Lateral **A**, **B** postoperative radiographs

The final reconstruction was performed by fixing the graft with six self-tapping cortex screws (4.5 mm) in the diaphysis part and three self-drilling cancellous bone screws (4.5 mm) in the epiphyseal part, all with a postero-anterior course: half of these with a medial to lateral direction and the other half with the opposite direction (Fig. 4).

The whole process lasted almost 5 h due to a considerable shortening of the preparation time of the bone graft: tumor and graft resections lasted about 30 min thanks to cutting guides.

Definitive histology confirmed the diagnosis and reported wide margins.

Immediately after the surgery, the patient showed a nerve palsy in the territory of the Common Peroneal Nerve, with the impossibility of dorsiflexing the toes and the right foot and a related sensitivity deficit. He was immediately treated with neurotropic supplements and electrostimulation of the muscles involved with progressive improvement of sensibility and movement.

After the surgery, weight-bearing was not allowed on the affected limb for two months; a brace to keep the knee full extension was used during the first month and then removed, allowing free knee mobilization, and then a rehabilitation procedure was set up to recover the knee's full range of motion.

After two months, the first signs of bone integration of the graft could already be detected by X-ray evaluation; a progressive load was also granted until complete weight-bearing in six months.

A three-month follow-up was set, with X-rays of the left knee and chest and knee CT scans with subtraction of artifacts.

After nine months, the X-rays showed that the graft was almost wholly fused to the patient's bone.

**Fig. 5:**
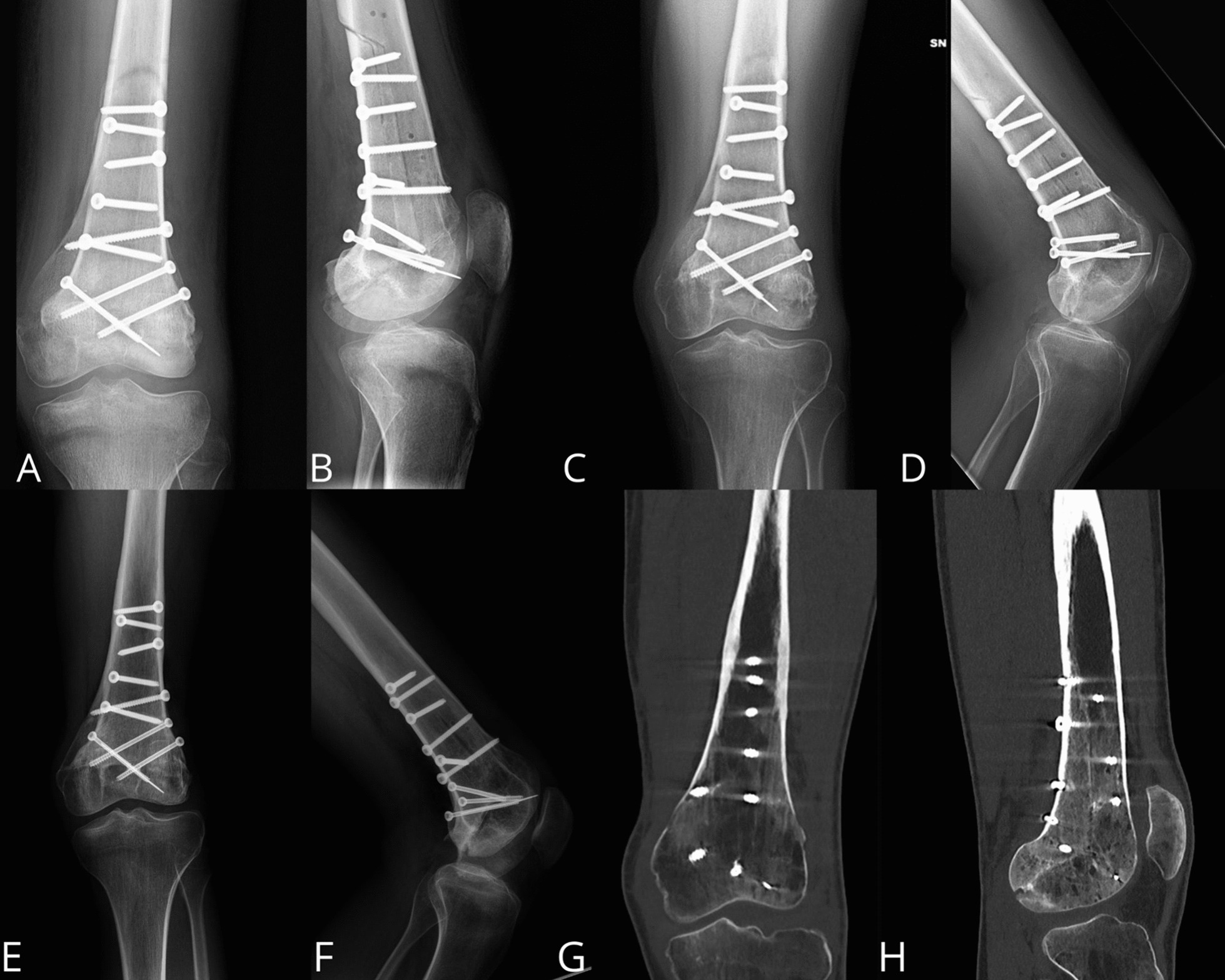
**A**, **B**: Antero-Posterior and Lateral X-rays at 3 months; **C**, **D**: Antero-Posterior and Lateral X-rays after 9 months: the graft is almost completely fused to the host bone; **E**, **F**: Antero-Posterior and Lateral X-rays at 12 months: the graft is perfectly integrated; **G**, **H**: CT scan at 18 months, the graft is perfectly integrated

At the last follow-up, 18 months after surgery, the X-rays showed the graft perfectly integrated, without signs of mobilization or recurrence. The patient walked without aids with full weight-bearing on the treated limb; the knee range of motion is complete in extension with a slight deficit in flexion of about 10°. The patient also regained the sensitivity at the level of the territory of the Common Peroneal Nerve with complete extension of the toes, even against resistance. Functional results were excellent, with an MSTS score of 30. (Fig. 5).

## Results

The research strategy returned 12 studies with a total of 151 patients affected by PO. The distal femur was the most frequently reported site (81.5%), followed by the proximal humerus (9 cases, 6.2%), the proximal tibia (4 cases, 2.75%), and the proximal femur (4 cases, 2.75%). The mean age of patients in all sites was 29 years old (range 25–31 years old). The mean follow-up of patients was 6.7 years (range 2.8–8.5 years) (Table 1).

**Table 1:** Systematic literature review about parosteal osteosarcoma in all sites between 1990 and 2022

Paper	All cases	Sites	Distal femur	Mean age	Mean FU (years)	Dediflcrentiation
Kavanagh et al. [20]	20	DF14;PH3;PF2;DR1	14	25.3	9.7	u.a.
Lewis et al. [16]	6	DF6	6	32	43	n.a.
Deijkers et al. [17]	6	DF5; FD1	5	29.7	5.4	n.a.
Hoshi et al. [21]	9	DF9	9	30.8	9.6	n.a.
Han et al. [5]	21	DF15;PH2;DT1;PF1;1L1; Pfil	15	25.5	92	14%
Agarwal et al. [19]	8	DF6;HS1;DR1	6	25.3	3.4	n.a.
Funovics et al. [9]	28	OT19:PH4:PF1:PT3:FS1	19	28.4	95	3.6%
Liu et al. [16]	13	DFI3	13	26.5	8.5	0
Nouri et al. [23]	11	DF8;FSI;DT1;PTI	8	25	2.8	54.6%
Wilke et al. [24]	12	1)1 12	12	27.3	/	n.a.
Prabowo et al. [8]	6	DF5;DH1	5	25.6	3	16%
Savvidou et al. [19]	11	DFI1	11	29	4.5	n.a.
Total	151	rjF12.3;PH9;PT4; DT2;FS2; PF4;IL1;DHI,DR2;HS1; Pfil	123	28.7	6.7	

(DF = Distal femur, FS = Femoral shaft, DT = Distal tibia, PT = Proximal tibia, PH = Proximal humerus, PF = Proximal femur, IL = Ileum, DH = Distal humerus, DR = Distal radius, HS = Humeral shaft, Pfi = Proximal fibula, n.a. = Not available)

After excluding different sites, 123 distal femur PO were analyzed. (Table 2).

**Table 2:** Systematic literature review about distal femur parosteal osteosarcoma between 1990 and 2022

Paper	Distal femur	Resection only	Cortical resection + graft	Prosthesis	Resection + Cementing	Knee arthrodesis	Rotationplasty/amputation	Margins	Local reoccurance	Complications/additional Surgery	MSTS (%)
Kavanagh et al. [20]	14			14				/	4	7(1 infection. 5lR, 1 loosing)	/
Lewis et al. [16]	6		6					5W, 1M	0	0	/
Deijkers et al. [18]	5		5					2w, 3M	0	3(2 failure, 1 graft future)	80
Hoshi et al. [22]	9		3	6				9W	0	3(2 TKA revisions/1 autograft fracture	83.7
Han et al. [23]	15	3		9	1	1	1	2IL, 4M, 9W	2	5 (3 revision, 1 fixation, 1 re-resection)	/
Agarwal et al. [18]	6		6					/	0	1 (graft fracture)	98.3
Funovics et al. [9]	19		7	7			4	2IL, 17W	3	7(3 single, 4 multiple)	80.6
Liu et al. [15]	13		13					/	1	1 (TKA for local reoccurance)	88.6
Nouri et al. [22]	8		1	4		2	1	1 IL, 7W	3	2(amputation for LR)	/
Wilke et al. [23]	12		7	5				12W	/	4(3 revision, 1 amputation for LR	76.6
Prabowo et al. [8]	5	1		2		1	1	5W	0	1 (revision for infection)	/
Savvidou et al. [19]	11		11					11W	1	1 (fibula amogtaft for LR)	93.5
loial	123	4	59	47	1	4	7	77W	14		85.9

(IL = Intralesional, W = Wide, M = Marginal, LR = Local recurrence, TKR = Total knee reconstruction)

The surgical treatment was resection of the posterior cortex of the distal femur and reconstruction with graft (26 autografts and 33 allografts) in half of cases (59/117 cases, 50%). The second most frequent type of surgery was distal femur resection and prosthetic reconstruction (47/117, 40%). Unexpectedly, 7 cases of amputation or rotationplasty and 4 knee arthrodesis were reported, especially in older cases with more complex management. (Table 2).

Margins were wide in 85.5% of reported cases (77/90 cases) independently from the types of surgery. (Table 2).

The total incidence of local recurrence was 11.4% (14/123 cases), but it was impossible to determine if there was a correlation with the types of surgery or margins obtained. (Table 2).

Functional results, evaluated with the MSTS score, were described in 7/12 papers; overall functional results were excellent in all cases, with a mean score of 85.9% (Table 2).

## Discussion

Parosteal osteosarcoma arises from the metaphyseal region over the cortical bone surface: in 80% of cases, in the posterior surface of the distal femur, followed by the proximal humerus and proximal tibia [1–3].

Wide margins surgery is the only treatment for this type of osteosarcoma, with survival up to 90% at 5 years [9, 11, 15]. Local recurrence may occur when wide resection is inadequate or in the case of dedifferentiation [12, 13].

Local recurrence was highly related from extent of resection with an incidence of 8–88% [9]. The incidence of metastatic disease range from 1 to 22% and was mostly related to dedifferentiation, but not to medullary involvement [9].

Historically, the treatment of PO in the distal femur was the posterior diaphysometaphyseal hemiresection, according to Campanacci; however, several reconstructions are still used [7–10].

Treatment choice is based on local extension, proximity to neurovascular structures, joint invasion, and extent of medullary involvement [9].

The influence of medullary involvement on survival is debated; some authors reported that the invasion into the medullary canal was a sign of tumor aggressiveness and local recurrence, but other authors have found no connection between medullary involvement and aggressiveness [14, 15].

Funovics et al. [9] and Liu et al. [12] compared biologic and prosthetic reconstruction after the resection of 28 PO in all sites, concluding that there is no difference between these two types of surgery in terms of local recurrence, metastases, or functional results; although they reported a higher incidence of revisions after prosthetic reconstructions.

Other authors reported satisfactory oncologic and functional outcomes without complications using the classic hemicortical resection technique with a 100% fusion rate of the allograft within 30 months postoperatively [12, 16–19].

The most common complication is host bone fracture, followed by local recurrence, nonunion, and infection. Rarely allograft fractures can occur. These complications are related to the size of the bone defect and often require surgical reintervention [14].

The incidence of nonunion for intercalary reconstructions with an allograft have been cited about 30% and fracture about 18% [17, 18].

Using the jigs, it is possible to spare more bone of the patients and make more accurate cuts; these allowed to reduce the nonunion and fracture rate. For these reasons, with the use of the jigs, theoretically, a plate is not needed as first step because the risk of fracture is lower.

Considering the good functional results and fast recovery to normal activities with prosthetic reconstructions, are biological reconstructions still indicated in these cases?

This literature review confirmed that the most widely used type of reconstruction remains the biological reconstruction with graft (47.9%), equally distributed between autologous grafts or allografts. In 40% of cases, distal femur resection and prosthetic reconstruction were reported. The only contraindication the hemiresection is the medullary canal involvement.

To improve the matching between the host bone and the graft and consequently the fusion of the graft, newer techniques with 3D printing technology can significantly influence the surgery.

In recent years, the techniques of designing and manufacturing surgical guides have been improved, as well as the printing material. With the rapid emergence of 3D printing technology, surgeons have started to apply this in nearly all areas of orthopedic surgery [23]. The orthopedic field that has benefited most from the development of 3D technologies is undoubtedly the oncology one [24, 25].

In 2021, Wu conducted a technical report of hemicortical bone tumor resection in the distal femur using a 3D printing guide plate for low-grade bone sarcomas [25]. In that study were used inactivated autologous bone grafts and reimplanted with plates and screws. This technology allowed surgeons to achieve negative resection margins more easily and safely, reducing surgical time and sparing important anatomic structures, leading to significant functional and reconstructive advantages for the patient [25–27].

We reported a case treated with the posterior diaphysometaphyseal hemiresection and reconstruction with allograft with custom jigs to guide both host and graft osteotomies. First, cutting guides allowed us to prepare the graft in a few minutes more safely and accurately, reducing the fracture risk, which could compromise the surgical procedure.

Second, through this procedure, we obtained a remarkable graft fitting to the host bone: only a minimal discrepancy remained at the level of the condyles due to minimal different anatomies. This fitting resulted in a considerable contact area, facilitating the integration.

Currently, the use of 3D cutting procedures has two types of limitations: time and cost. The time required to develop and produce all the projects is currently about three to four weeks and should be further reduced in order to be able to develop a similar plan even in cases of much more aggressive sarcomas. The cost to produce 3D printed cutting guides is company and material dependent and may be variable in different countries.

In conclusion, in patients with PO, the classic posterior distal femur diaphysometaphyseal hemiresection and reconstruction with allograft is still indicated due to the low grade of the tumor, the young age of the patients, good functional results, and lower incidence of complication than prosthetic reconstructions. The 3D-printed cutting guides have many advantages: precise cuts permit a reduction of surgical time, improving accuracy of negative surgical margins, sparing healthy tissue, and reducing the risk of graft breakage during preparation. In the same time, they lead to a better fit between graft and host bones with a consequent reduction in the integration time.

## Data Availability

Information about clinical, imaging and histological details were found in the clinical chart of the patient of patients were contacted by phone.
